# Inhaled liposomal ciprofloxacin protects against lethal tularemia in the common marmoset

**DOI:** 10.1128/aac.01232-25

**Published:** 2025-12-19

**Authors:** Rachel E. Ireland, Alejandro Nunez, Wendy Butcher, Carwyn Davies, James D. Blanchard, Francis Dayton, Igor Gonda, Sarah V. Harding, Michelle Nelson

**Affiliations:** 1 CBR Division, Defence Science and Technology Laboratory (Dstl) 13330, Salisbury, Wiltshire, United Kingdom; 2 Animal and Plant Health Agency 16232, Addlestone, Surrey, United Kingdom; 3 Aradigm Corporation 300404 , Hayward, California, USA; City St George's, University of London, London, United Kingdom

**Keywords:** animal model, tularemia, inhalation

## Abstract

*Francisella tularensis* is a gram-negative, intracellular bacterium that causes the disease tularemia. Tularemia is prevalent in North America, Europe, and Asia and is typically treated with injected and orally administered antibiotics, including streptomycin, gentamicin, doxycycline, and ciprofloxacin, administered for 10 to 21 days. New therapeutic options are required to reduce the potential of a relapse of disease. Inhaled liposomal-encapsulated ciprofloxacin has demonstrated protection in a murine model of tularemia. The efficacy was further assessed in a nonhuman primate model of tularemia. Mixed-sex common marmosets were challenged with *F. tularensis* by the inhalational route, and the efficacy of ciprofloxacin delivered by either the inhalational (Apulmiq liposomal formulation) or oral route was compared. Antibiotics were initiated either at 24 h post-challenge (post-exposure prophylaxis) or at the onset of fever (treatment) and continued for 7 days. All control (untreated) animals succumbed to infection by 8 days post-challenge. All animals that received antibiotics, by either route, survived the duration of the study, with bacterial clearance in all but one animal that received inhalational ciprofloxacin. Antibiotic treatment also reduced the physiological and immunological responses observed when compared to animals that received no antibiotics. Histological changes in the lungs were less frequent, although mild, resolving lesions were present in animals treated with ciprofloxacin delivered at the onset of fever by either route.

## INTRODUCTION

*Francisella tularensis* is the causative agent of the disease tularemia, which is prevalent in North America, Europe, and Asia (1). Human disease can be the result of infection by a number of routes: through arthropod bites or skin wounds, through the conjunctiva, by eating or drinking contaminated substances, or via inhalation of bacterial particles (2). There are six classical forms of tularemia: ulceroglandular, glandular, oropharyngeal, and oculoglandular, with the typhoidal and pneumonic forms being the most severe (3). Pneumonic disease can be secondary (and develop as a consequence or complication of other forms of disease) or primary following inhalational exposure. Infection acquired by the inhalational route has an incubation period typically between 3 and 5 days, initially presenting with generic febrile, flu-like symptoms, which may progress to sepsis or pneumonia and may be lethal (3, 4). Streptomycin, gentamicin, doxycycline, or ciprofloxacin administered for 10 to 21 days is recommended for the treatment of pneumonic tularemia, with doxycycline or ciprofloxacin recommended for post-exposure prophylaxis (PEP) and delivered orally or by injection (1). However, factors such as the delay in treatment and disease complications, such as lymph node enlargement, can change or increase the duration of treatment (5). Disease relapse can occur even when treated with the recommended antibiotics (6, 7); therefore, it is prudent to explore alternative antibiotics and/or alternative delivery strategies.

Several alternative antibiotics have been explored in animal models in recent years, including in nonhuman primates (5, 8–13). Exploration of alternative delivery routes, such as inhaled delivery, is less common. There are currently four inhaled drugs licensed for chronic bacterial infections in cystic fibrosis patients: aztreonam lysine, colistin, levofloxacin, and tobramycin (14–16). Inhaled liposomal amikacin has been approved for the treatment of respiratory infections caused by the *Mycobacterium avium* complex and also has potential for intensive care patients (17, 18). The use of inhaled drugs to treat acute infections is not widely reported, although one group suggests that lung-targeted, low-dose antibiotic regimens delivered by the inhalational route may be helpful in combating antimicrobial resistance (19).

Two inhaled forms of ciprofloxacin have been assessed for a number of acute bacterial infections, including tularemia, in murine models of infection (20–23). These liposomal-encapsulated formulations (Lipoquin and Apulmiq) were developed by Aradigm Corporation (24, 25). They offer several advantages over oral formulations, as they can prevent dissemination of bacteria by targeting the infection while disease is localized in the lung. In addition, the low doses delivered locally to the respiratory tract could prevent antibiotic-associated systemic adverse effects. In the murine model of pneumonic tularemia, a single inhaled dose was shown to be more efficacious when compared with orally delivered ciprofloxacin (20). The pharmacokinetics of Apulmiq in the marmoset has previously been determined and indicates once-daily dosing would maintain the concentration of ciprofloxacin in the plasma and the lungs, above the minimum inhibitory concentration (MIC) of 0.03 µg/mL for *F. tularensis* (26). This dosing regimen also ensures the AUC is within an equivalent range that would be obtained in humans following dosing and that was efficacious in the murine model.

The two formulations, Lipoquin (50 mg/mL of ciprofloxacin hydrochloride salt, with more than 99% encapsulated in liposomes) and Apulmiq (a 1:1 mix of Lipoquin with a non-liposomal solution of 20 mg/mL free ciprofloxacin), have also been evaluated in humans (25, 27). Apulmiq was found to be effective at reducing pulmonary exacerbations in a randomized, double-blind, placebo-controlled phase 3 clinical trial (ORBIT-4) in non-cystic fibrosis bronchiectasis patients with chronic *Pseudomonas aeruginosa* infections. The wealth of safety data makes it an attractive product for repurposing for other indications. The efficacy of Apulmiq was determined in the New World nonhuman primate, the common marmoset (*Callithrix jacchus*), following infection with *F. tularensis*.

## RESULTS

### PEP use of inhaled ciprofloxacin is completely efficacious against inhalational tularemia

A cohort of 16 marmosets was challenged, in pairs, with a mean dose of 462 ± 63 CFU of *F. tularensis* by the aerosol route. There were no significant differences between the challenge doses received by the different groups of animals (*P* = 0.92). At 24 h post-challenge, four animals received either Apulmiq (0.8 mg/kg) or empty liposomes (i.e., without antibiotic) by the inhalational route. A group of four animals received ciprofloxacin (50 mg/kg) by the oral route, and a group remained untreated. The dosing continued for 7 days, either daily (Apulmiq or empty liposomes) or at 12-hourly intervals (oral ciprofloxacin) to ensure equivalence to human dosing regimens.

**Fig 1:**
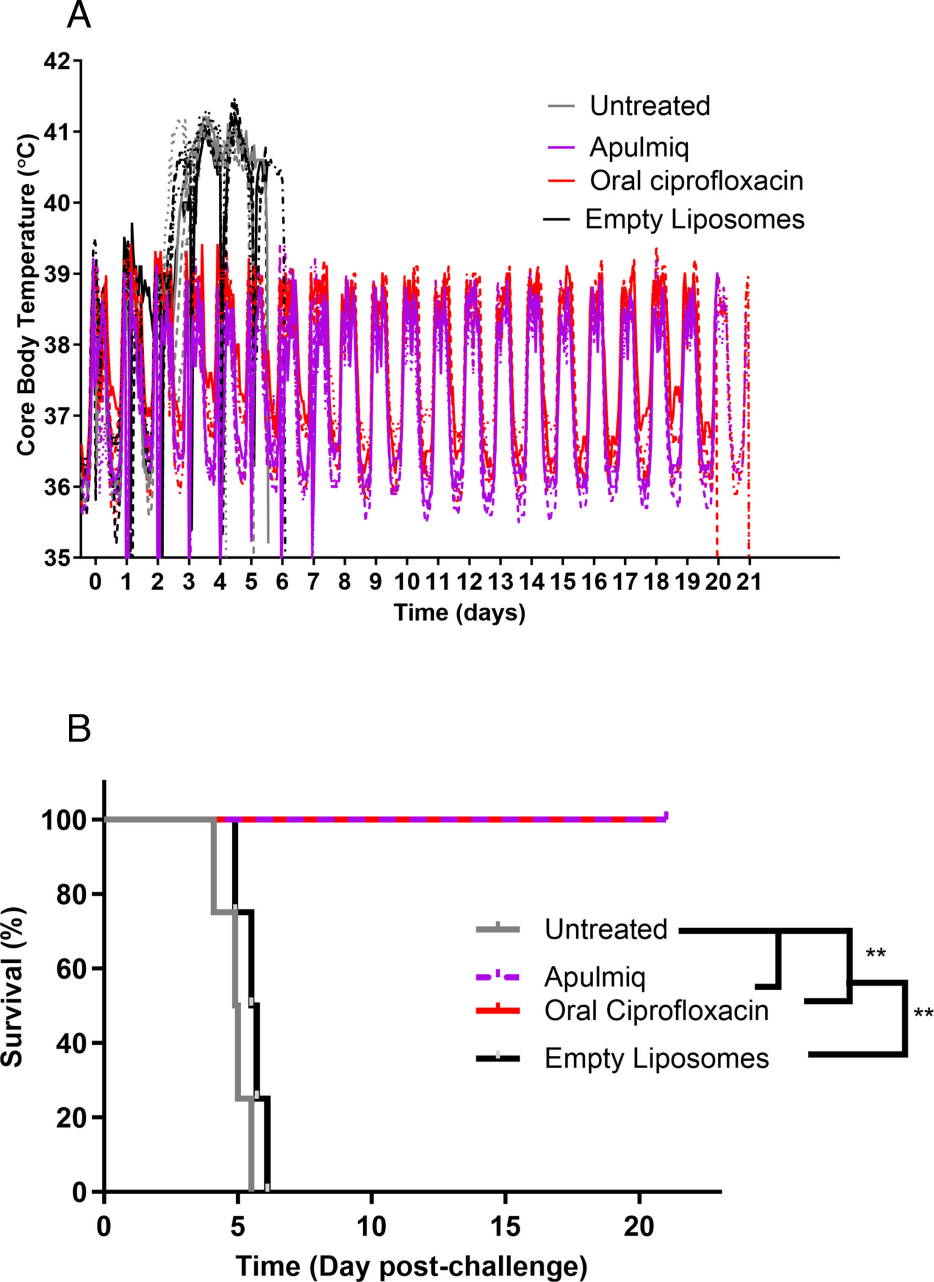
The temperature profile (**A**) and survival (**B**) of marmosets challenged with *F. tularensis* by the inhalational route and treated with PEP antibiotics at 24 h post-challenge. Survival analysis was performed using log-rank Mantel-Cox tests to test for significant differences in survival where **P < 0.01.

Animals that did not receive treatment or received empty liposomes became febrile, as defined by a temperature of >1.5°C above the normal daytime temperature of 38.6 ± 0.3°C or the nighttime temperature of 36.4 ± 0.5°C (Fig. 1A). The mean time to become febrile was 57.7 ± 1.7 h post-challenge. This ranged from 55.1 to 57.3 h in the group that received no treatment, and 57.7 to 59.8 h in the group that received empty liposomes. Mild clinical signs were observed approximately 24 h after the onset of fever, starting with animals becoming more subdued, progressing to a ruffled coat and a slightly hunched posture, with a slight reddening of the face at the humane endpoint. Animals were euthanized if they had any clinical signs when the temperature declined to 39°C following the onset of fever. Animals that did not receive antibiotics were euthanized at a mean time of 4.9 days post-challenge for animals that received no treatment or 5.5 days for animals that received empty liposomes (Fig. 1B). Animals that received either ciprofloxacin orally or by the inhalational route (Apulmiq) did not develop fever (Fig. 1A) or clinical signs of disease and survived until the end of the study.

### Inhaled ciprofloxacin was efficacious against inhalational tularemia when administered at the onset of fever

**Fig 2:**
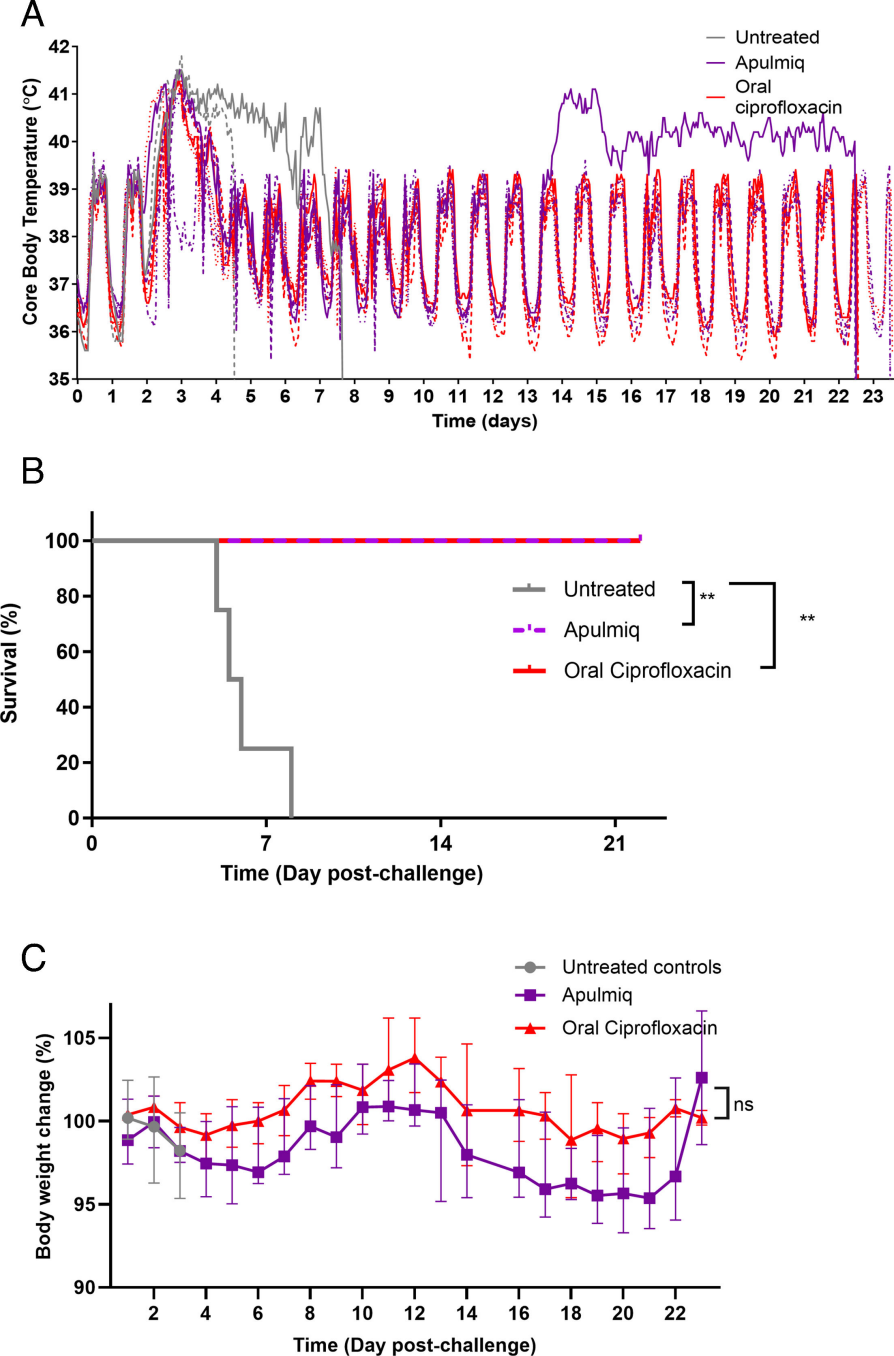
The temperature profile (**A**), survival (**B**), and weight (**C**) of marmosets challenged with *F. tularensis* by the inhalational route and then treated with antibiotics at the onset of fever (treatment). Survival analysis was performed using log-rank Mantel-Cox tests to test for significant differences in survival where **P < 0.01. Weight changes were assessed using two-way ANOVA.

A cohort of 12 marmosets was challenged with *F. tularensis* by the aerosol route and received a mean challenge dose of 216 ± 48 CFU. All animals became febrile between 57 and 70 h post-challenge (Fig. 2A). Therapy was initiated once all animals in the treatment group were febrile. Animals received inhalational Apulmiq (0.8 mg/kg lung dose; once daily) or ciprofloxacin delivered orally (50 mg/kg; twice daily) for 7 days. Control animals received a media-only placebo twice daily. These animals remained febrile and developed overt clinical signs, including a subdued nature, ruffled coat, a slightly hunched posture, and a reddening of the face at the humane endpoint. These animals were euthanized following a decline in temperature between 4.8 and 7.7 days post-challenge (Fig. 2B). Animals that received either Apulmiq or oral ciprofloxacin resolved their fever within 3 to 46 h of the initiation of therapy, with a complete return to their normal diurnal rhythm within 53 to 81 h. The temperature profile of all animals remained within their normal diurnal rhythm until the end of the study, except for one animal (Apulmiq treatment group) that developed a secondary fever on day 13 post-challenge. The temperature for this animal remained elevated until the end of the study, although no clinical signs of disease were observed. All treated animals survived until the end of the study, the administration of both Apulmiq and oral ciprofloxacin providing a protective benefit (*P* = 0.0067, for both). Animals lost up to 5% of their body weight during the first 6 days post-challenge, following which they steadily regained this weight until day 12 post-challenge (Fig. 2C). Between days 12 and 20 post-challenge, further weight loss occurred, peaking at ~5%, which was steadily regained until the end of the study. There were no differences between the weight loss profiles of marmosets receiving either Apulmiq or oral ciprofloxacin (*P* = 0.1748) or between the pre-challenge and post-mortem weights for antibiotic-treated or untreated marmosets (*P* > 0.05) (Fig. 2C).

### Antibiotic treatment resulted in bacterial clearance in most animals

**Fig 3:**
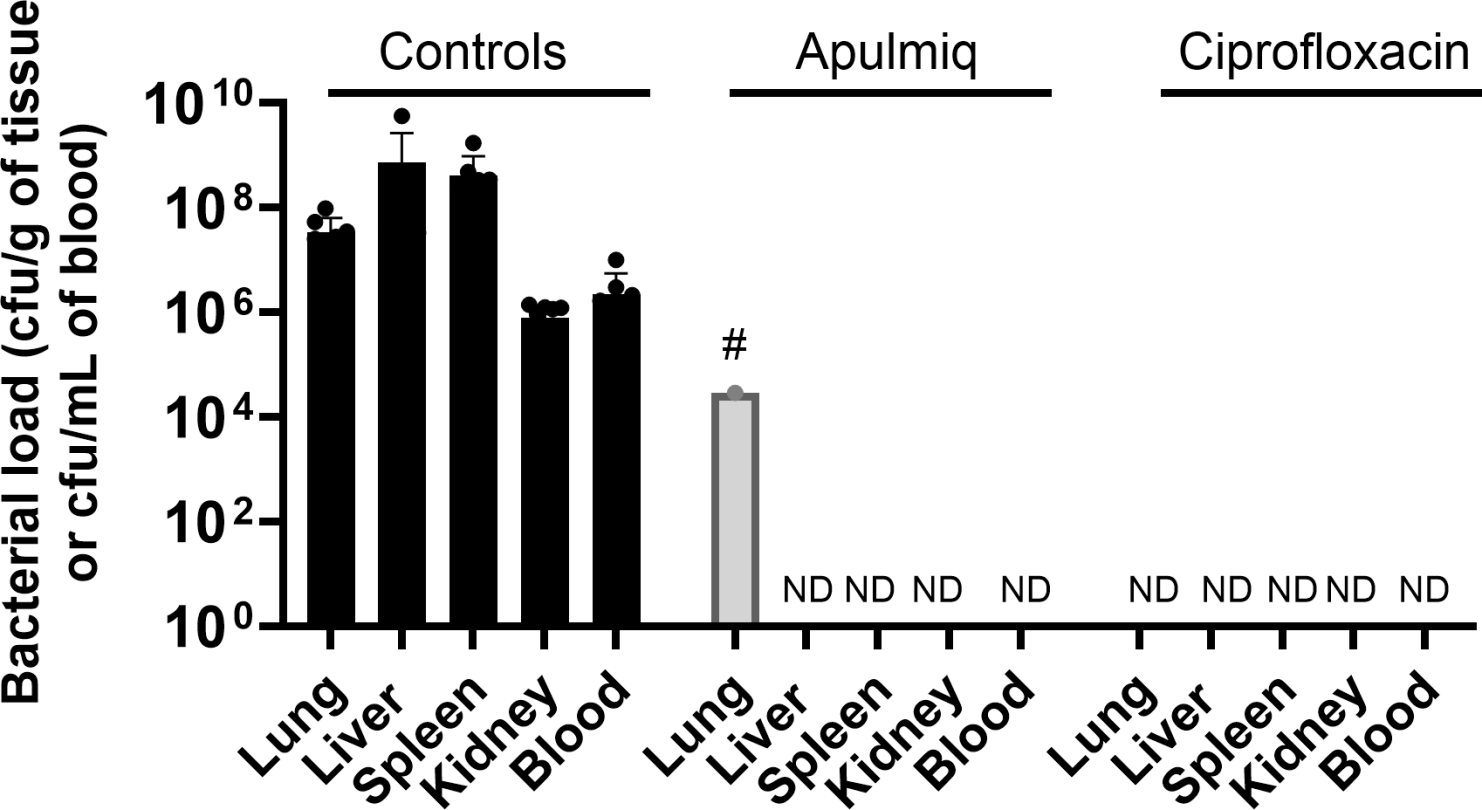
The bacterial load isolated from a panel of tissues and blood following challenge with inhalational *F. tularensis* and antibiotic treatment (both the PEP and treatment studies). Tissues were homogenized, serially diluted in phosphate-buffered saline, and sub-cultured onto blood cysteine glucose agar plates. Counts are expressed as colony-forming units per gram of tissue or colony-forming units per milliliter of blood and are presented as the mean data and the standard error of the mean for each treatment group. ND, bacteria not detected. #, Bacteria were only present in one of the four animals.

Tissues and blood were assessed for the presence of *F. tularensis* when animals succumbed to disease or were euthanized at the end of each study. In both the PEP and treatment studies, high levels of bacteria were present in the lungs, liver, spleen, and kidneys harvested from all the untreated animals (animals that received no treatment, placebo, or empty liposomes) (Fig. 3). The highest concentration of bacteria was detected in the liver and spleen, with a mean of 7.3 × 10^8^ CFU/g of tissue (ranging between 1.2 × 10^4^ and 5.6 × 10^8^) and 4.2 × 10^8^ CFU/g of tissue (ranging between 9.2 × 10^5^ and 1.7 × 10^9^), respectively. Bacteremia was also evident in these animals, with a mean of 2.3 × 10^6^ CFU/mL of blood (range of 2.5 × 10^1^ to 1.0 × 10^7^). No bacteria were recovered from the tissues or blood of animals treated with Apulmiq or orally delivered ciprofloxacin, with the exception of one animal treated with Apulmiq at the onset of fever. That animal had 2.9 × 10^4^ CFU/g of *F. tularensis* detected in the lungs. This was the animal that also developed a secondary fever. *F. tularensis* could not be detected in other tissues collected from that animal, even following enrichment of the remaining organ homogenates in liquid media. The MIC of selected colonies isolated from the lung of that animal was comparable to wild-type *F. tularensis* Schu S4 (0.032 µg/mL). Therefore, the relapse of infection due to the development of resistance to ciprofloxacin in this animal can be discounted.

### Antibiotic treatment reduced the physiopathological response to infection

**Fig 4:**
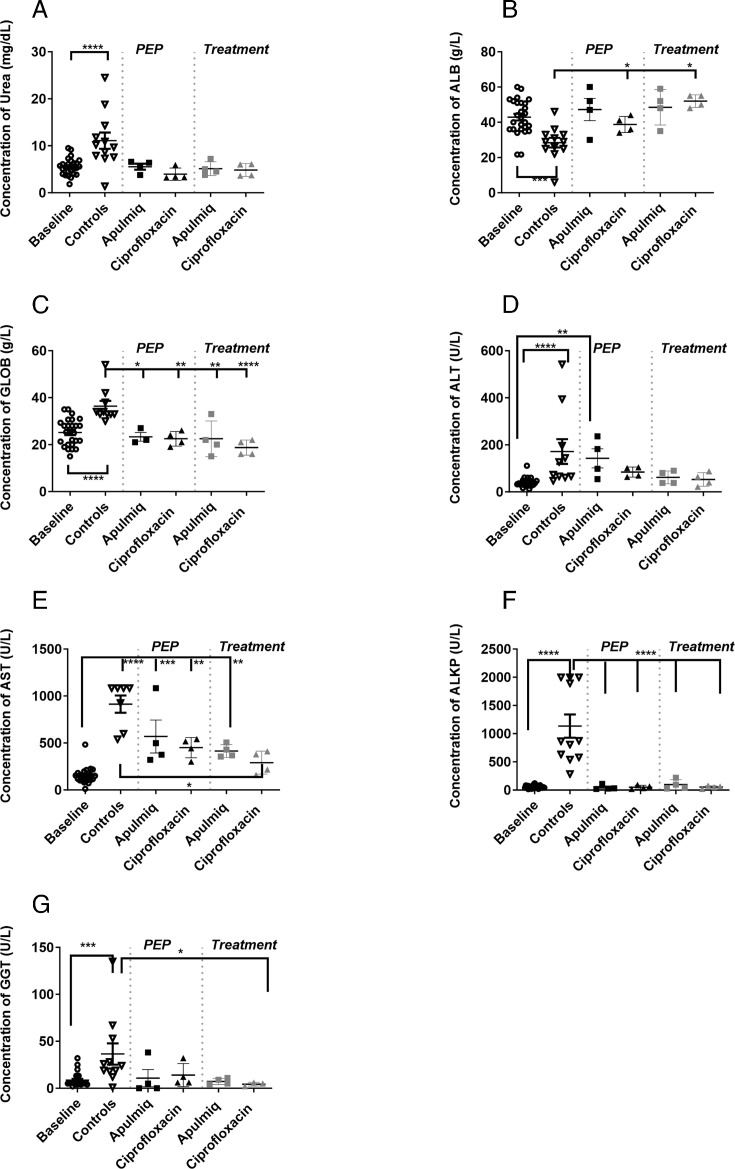
Clinical chemistry parameters observed in marmosets challenged following challenge with inhalational *F. tularensis* and administered antibiotics. (**A**) BUN (mg/dL), (**B**) ALB (albumin, g/L), (**C**) GLOB (globulin, g/L), (**D**) ALT (U/L), (**E**) AST (U/L), (**F**) ALKP (U/L), (**G**) GGT (U/L). Analysis was performed by one-way ANOVA of the log-transformed data, where * is *P* < 0.05, ** is *P* < 0.01, *** is *P* < 0.001, **** is *P* < 0.0001. Baseline data were collected from all animals prior to challenge and combined irrespective of which treatment group they were then allocated.

There were significant changes in a number of clinical chemistry parameters in animals that received no treatment (animals that were untreated or received placebo or empty liposomes) at the time of euthanasia compared to the baseline values (Fig. 4). This included a significant increase in the levels of blood urea nitrogen (BUN; *P* < 0.0001), globulin (*P* < 0.0001), alanine transaminase (ALT; *P* = 0.003), aspartate aminotransferase (AST; *P* < 0.0001), alkaline phosphatase (ALKP; *P* < 0.0001), and gamma-glutamyl transferase (GGT; *P* = 0.0007). In addition, there was a significant reduction in the levels of serum albumin (*P* = 0.0001). In general, the administration of antibiotics to animals maintained the levels of these parameters within baseline measures. However, there was some evidence of liver dysfunction, with a significant increase in the level of ALT in animals that received Apulmiq prophylactically (*P* < 0.01), and in the levels of AST in all animals that received Apulmiq (*P* < 0.001 for PEP and *P* < 0.01 for treatment) and those that received ciprofloxacin (*P* < 0.01) prophylactically.

### Antibiotic treatment prevented severe histological changes in tissue, although mild pathology was observed in the lungs of some animals treated with ciprofloxacin

**Fig 5:**
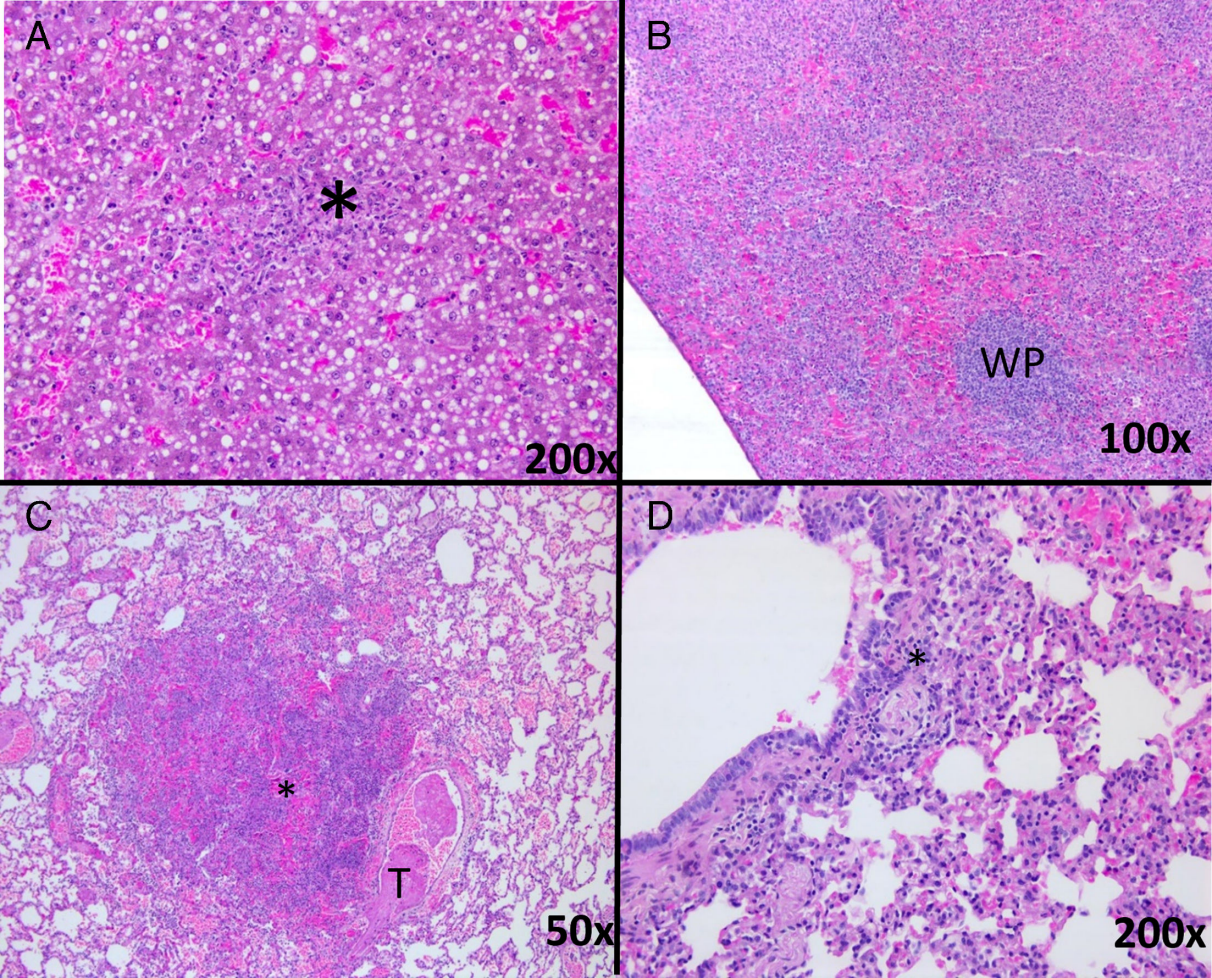
Representative histological images from marmosets challenged by the inhalational route with *F. tularensis*. (**A**) Liver from an animal administered empty liposomes post-challenge indicating a minimal severity, focal lesion in the liver, with tissue destruction and leukocyte infiltration. (**B**) Spleen from an animal that received empty liposomes post-challenge indicating a severe, extensive lesion in the red pulp with lymphocytic areas in white pulp (W) still identifiable. (**C**) Lung from an untreated animal showing moderate typical pulmonary lesions with inflammatory exudate in the central area and a fibrin thrombi (T) visible in a blood vessel. (**D**) Lung from an animal given Apulmiq indicating a thickened alveolar septa with histiocytic and lymphocytic infiltration and an accumulation of lymphocytes close to a bronchiole and around a blood vessel (*).

Animals that did not receive antibiotics had extensive histopathological lesions in the lung and spleen typical of inhalational tularemia, with less extensive lesions in the liver and occasionally in the lymph nodes (Fig. 5). Typical lesions observed in the liver of these animals were multifocal acute necrotizing and neutrophilic hepatitis of minimal to mild severity (Table 1). In the spleen, minimal to severe random multifocal to diffuse acute fibrin necrotizing and neutrophilic splenitis (in red pulp) was observed, with rare vascular thrombosis and acute fibrin necrosis. Mild to severe neutrophilic (broncho)pneumonia with perivascular edema was observed in the lungs, with frequent thrombosis (thromboembolic events) in blood vessels. Alveolar hemorrhage was evident in one animal.

**TABLE 1:** Summary of the lesion severity in tissues harvested from marmosets challenged with *F. tularensis* and then administered antibiotics (Apulmiq or ciprofloxacin) either as PEP (24 h post–challenge) or treatment (onset of fever)

Tissue	Lesion severity/other feature	Controls^*a*^	PEP		Treatment	
			Apulmiq	Ciprofloxacin	Apulmiq	Ciprofloxacin
		12 animals	4 animals	4 animals	4 animals	4 animals
Liver	No acute lesions	0	4	4	4	4
	Minimal	2	–^*b*^	–	–	–
	Mild	9	–	–	–	–
	Moderate	1	–	–	–	–
	Severe	0	–	–	–	–
Spleen	No acute lesions	0	4	4	4	4
	Minimal	2	–	–	–	–
	Mild	1	–	–	–	–
	Moderate	4	–	–	–	–
	Severe	5	–	–	–	–
Lung	No acute lesions	1	4	4	2	3
	Minimal	–	–	–	–	–
	Mild	3	–	–	1	–
	Moderate	7	–	–	–	–
	Severe	1	–	–	1	–
	Interstitial pneumonia	–	3	1	–	–
	Hemorrhages	1	–	–	–	–
	Chronic lesion	–	–	–	1	1

^*a*^ Includes animals that may have been untreated, received placebo, or empty liposomes.

^*b*^ –, these animals do not present with this specific severity of the lesions.

No histopathological changes were observed in the tissues of six of eight animals treated with ciprofloxacin by either dosing route. One of the animals administered oral ciprofloxacin as PEP had minimal to mild interstitial pneumonia. This was also observed in three of the four animals treated with Apulmiq as a PEP. Mild acute lesions were observed in the lungs of two animals that had antibiotic treatment delayed until the onset of fever, one of which received oral ciprofloxacin and the other Apulmiq. Both these animals also had chronic or resolving lesions, suggesting that the lungs were undergoing repair.

Interestingly, one animal that received Apulmiq and had a secondary fever and bacteria detected in the lungs had severe and extensive pulmonary lesions, with dissemination of the lesions limited to the mediastinal (draining) lymph node.

### Immunohistochemical (IHC) analysis was used to characterize the lesions observed

**Fig 6:**
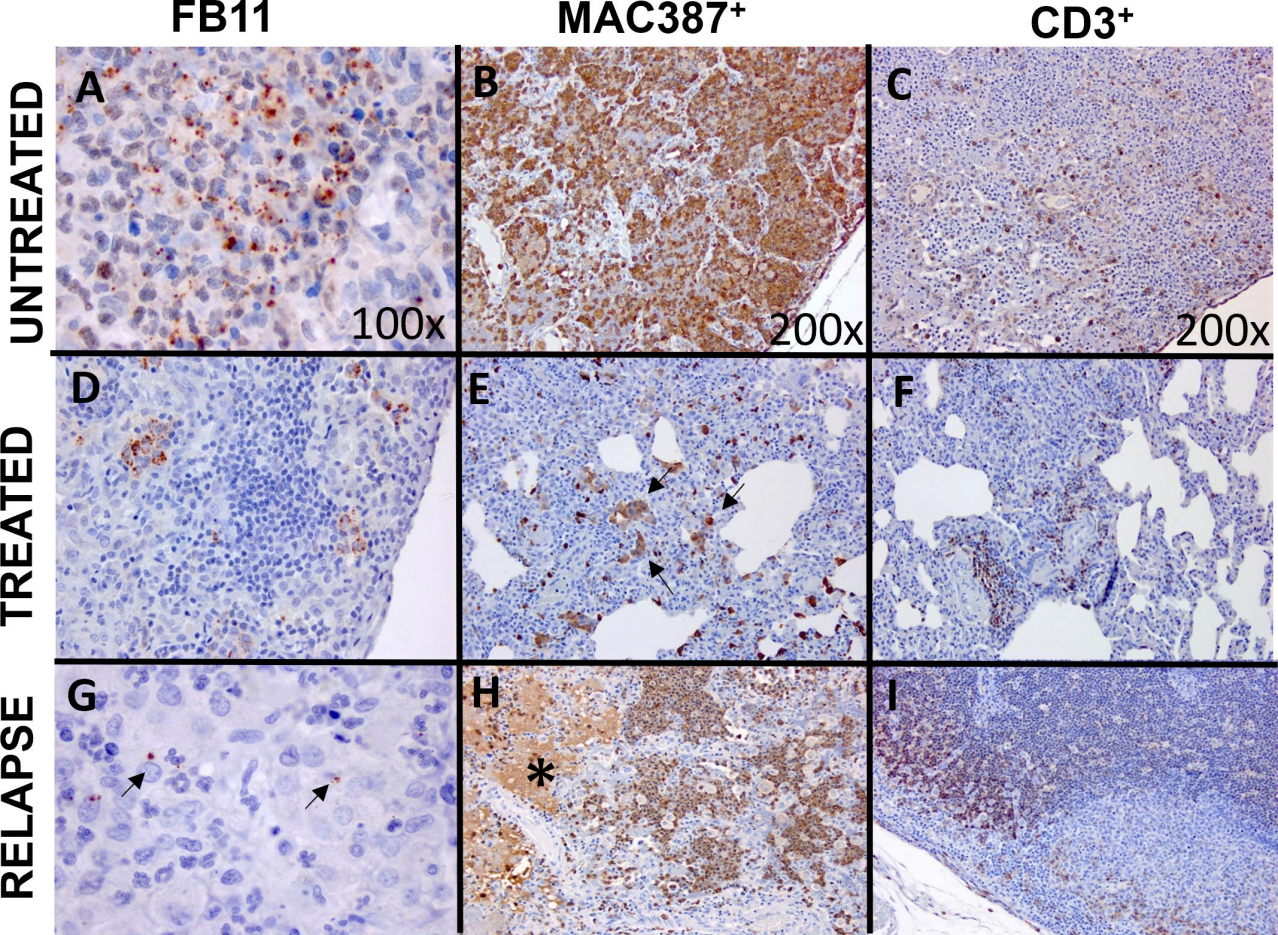
Representative stained lung section images from marmosets challenged by the inhalational route with *F. tularensis* and then administered placebo or antibiotics at the onset of fever. Lung tissue from placebo-treated animals was stained with FB11 (antibody used to detect the LPS of *F. tularensis*) (**A**), MAC387 antibody (macrophages/neutrophils) (**B**), and an anti-CD3 antibody (T cells) (**C**). Brown coccobacilli were located intracytoplasmically and extracellularly with abundant labeling of bacteria, neutrophils, macrophages, and T cells in areas of acute inflammation. Extracellular staining of neutrophils, macrophages, and T cells was also abundant in areas of exudation. Less bacterial staining was observed in the lung tissue of animals administered antibiotics (**D**, ciprofloxacin-treated animal) with MAC387^+^ (**E**, ciprofloxacin-treated animal) and CD3^+^ cells (**F**, Apulmiq-treated animal) and in areas of septal thickening and cellular infiltration. The arrows indicate stained clusters of macrophages. The animal that received Apulmiq and relapsed had some bacterial staining (**G**), with the arrows highlighting individual bacterial cells. Neutrophils and macrophages were observed in areas of acute inflammation (**H**), with areas of diffuse extracellular labeling (*) likely due to presence of cellular components and debris in the exudate. CD3^+^ cells were observed in the cortex (**I**).

Selected lung tissues from each group of animals in the treatment study (placebo, oral ciprofloxacin, and Apulmiq) were further characterized using IHC staining. Gram Twort staining and an anti*-F tularensis* LPS monoclonal antibody were used to assess the presence of bacteria and/or bacterial antigen (Fig. 6; Table 2). In both untreated animals, there was a low to moderate amount of Gram Twort staining of the lesions, both intracellular and extracellular. The more sensitive *F. tularensis* anti-LPS antibody detected moderate amounts of staining in both these animals (Fig. 6A). This was typically observed in macrophages within areas of inflammation and also in extracellular areas of fibrinous and necrotizing exudation. Intracytoplasmic labeling was also observed in macrophages in areas without visible acute inflammation. Bacteria or bacterial antigen was not detected in the lungs of two out of the four animals that received antibiotics (one that received oral ciprofloxacin and one that received Apulmiq). However, an occasional to a low level of staining was observed in the other two treated animals. One of these treated animals was the animal that received Apulmiq and developed a secondary fever (Fig. 6G). The other animal received oral ciprofloxacin (Fig. 6D) but had no evidence of any secondary disease. In both of these animals, there was bacterial staining observed in the intracytoplasmic regions of phagocytes and in areas of exudate mixed with cell debris in the animal that developed a secondary fever.

**TABLE 2:** Summary of the immunohistochemical staining levels in the lungs harvested from marmosets challenged with *F. tularensis* and then administered antibiotics (Apulmiq or ciprofloxacin) at the onset of fever^*a*^

	Stain					
Animal treatment group	H&E	Gram Twort	FB11	MAC387	CD3	CD79a
Untreated 1	+	+	++	++	+	−
Untreated 2	+++	++	++	+++	++	+/−
Apulmiq 1	+	−	−	+	++	+/−
^*b*^Apulmiq 2	+++	+	+	+++	++	+/−
Ciprofloxacin 1	−	−	−	+	+	−
Ciprofloxacin 2	+	+/−	+	++	++	−

^*a*^ −, negative; +/−, minimal/rare; +, mild low number; ++, moderate; +++, abundant.

^*b*^ This animal had a secondary fever.

The lesions were further characterized to identify the immune cells involved. Acute lesions in untreated animals had a moderate to abundant amount of staining of neutrophils/macrophages in the affected areas as well as in the exudate (Fig. 6B). One animal had a small amount of staining for T cells in the alveolar septa or in the alveoli in areas outside of the lesion (Fig. 6C), whereas the other animal had moderate staining in areas of cellular infiltration and in perivascular and peribronchial locations. The presence of B cells was also observed in this animal.

There was a small amount of staining for neutrophils/macrophages in animals that were successfully treated with antibiotics (one animal that received oral ciprofloxacin and one that received Apulmiq) (Fig. 6E). This was scattered in the septa or alveoli, with occasional accumulation in the peribronchial spaces or some septa. In the animal that received oral ciprofloxacin, there was also a small amount of staining for T cells, again scattered in the alveolar septa, with some clusters in the peribronchial locations, with no evidence of B cell staining. There was increased staining for T cells in the animal that had received Apulmiq (Fig. 6F), with occasional staining for B cells.

Two of the animals that received antibiotics had histopathological changes in the lungs (the animal that had received Apulmiq and presented with a secondary fever, and one that received oral ciprofloxacin and had no overt signs of infection). The animal that received oral ciprofloxacin had mild lesions, and the animal that received Apulmiq had severe lesions. Both animals had neutrophil/macrophage staining in the exudate, with occasional scattered staining in the septa and alveoli, although this was more abundant in the animal that had received Apulmiq (Fig. 6H). There was a moderate level of staining for T cells frequently observed in areas of cellular infiltration in perivascular and peribronchial locations (Apulmiq) (Fig. 6I) or in areas of septal infiltration (oral ciprofloxacin). Additionally, a minimal amount of staining for B cells was observed in the exudate for the animal that received Apulmiq.

### IgM, but not IgG, was detected in the blood of animals that survived at the end of the study

**Fig 7:**
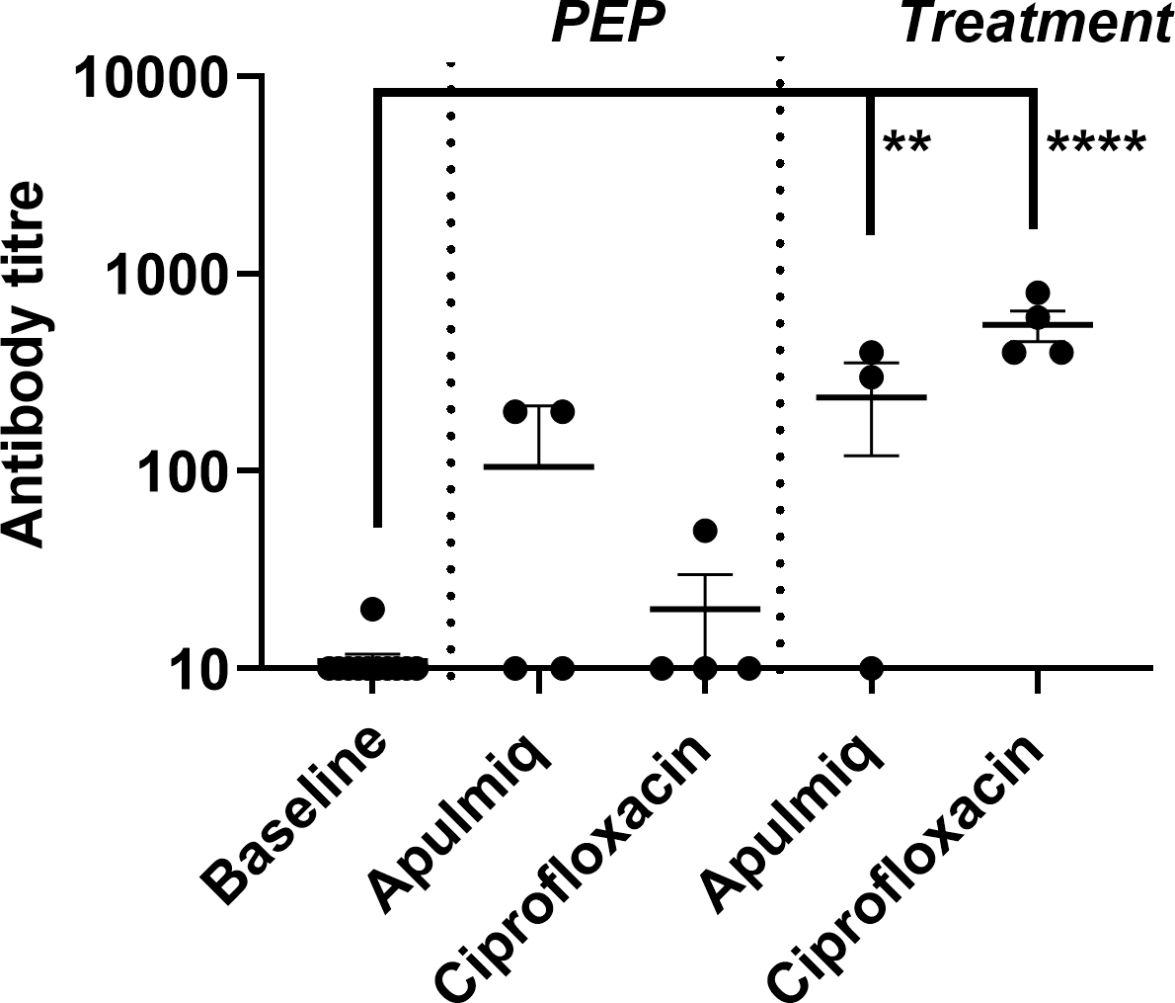
The level of IgM in the plasma of animals challenged with *F. tularensis* by the inhalational route and then administered antibiotics (Apulmiq or ciprofloxacin) either as PEP (24 h post-challenge) or treatment (onset of fever). Significant difference from baseline (pre-challenge samples) was determined using a two-way ANOVA with Tukey’s multiple comparisons test, where **P < 0.01 and ****P < 0.0001. Control animals were euthanized when they reached the humane endpoint (between day 4 and day 8 post-challenge), PEP and treatment animals were euthanized at the end of the study (day 21 and day 244, respectively).

The level of IgM in plasma was determined by ELISA at the end of both the PEP and treatment studies (Fig. 7). The level of detectable IgM was significantly higher in the animals that were treated at the onset of fever, compared to the baseline levels, than in those treated at 24 h post-challenge (*P* < 0.001 and *P* < 0.01, respectively). One animal that received Apulmiq following the onset of fever did not appear to have developed antibodies. The levels of IgG were also assessed, but no antibody was detected in any sample.

### Administration of antibiotics resulted in a reduced inflammatory cytokine response

**Fig 8:**
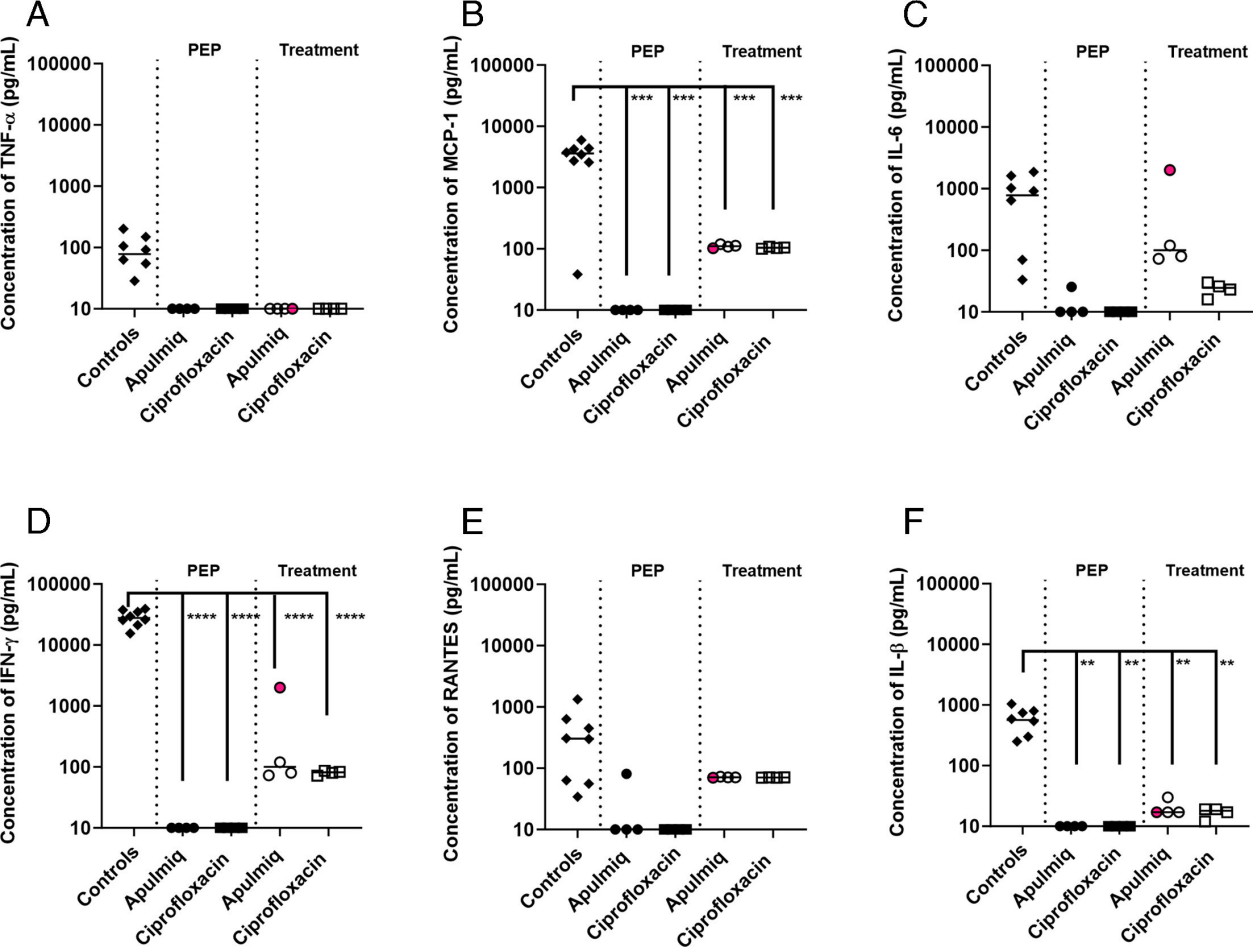
The level of selected cytokines in the plasma of animals that survived following challenge with *F. tularensis* by the inhalational route and administration of antibiotics (Apulmiq or ciprofloxacin) either as PEP (24 h post-challenge) or as treatment (onset of fever). (**A**) TNF-α, (**B**) MCP-1, (**C**) IL-6, (**D**) IFN-γ, (**E**) RANTES, and (**F**) IL-1β. Significant difference from control animals that received no antibiotics was determined using a two-way ANOVA with Tukey’s multiple comparisons test, where **P < 0.01, ***P < 0.001, and ****P < 0.0001. The highlighted pink data point represents the animals that relapsed following treatment with Apulmiq. Control animals were euthanized when they reached the humane endpoint (between day 4 and day 8 post-challenge); PEP and treatment animals were euthanized at the end of the study (day 21 and day 244, respectively).

Animals that did not receive antibiotics had high levels of TNF-α, MCP-1, IL-6, IFN-γ, RANTES, and IL-1β in the plasma at the time of euthanasia (Fig. 8). Animals that received oral ciprofloxacin or Apulmiq, either as PEP or treatment, had a reduced level of these cytokines at the end of the study (21–23 days post-challenge). Only one animal that received Apulmiq as PEP had a low level of IL-6 and RANTES; otherwise, there were no detectable cytokines in any animal that received either antibiotic as PEP. Animals that were treated following the onset of fever all had low levels of MCP-1, IL-6, IFN-γ, RANTES, and IL-1β, although no TNF-α was detected. The animal that received Apulmiq and had a secondary fever had higher levels of IL-6 and IFN-γ in the plasma compared with other treated animals. However, this level was approximately 10-fold lower than the levels detected in animals that did not receive antibiotic treatment.

**Fig 9:**
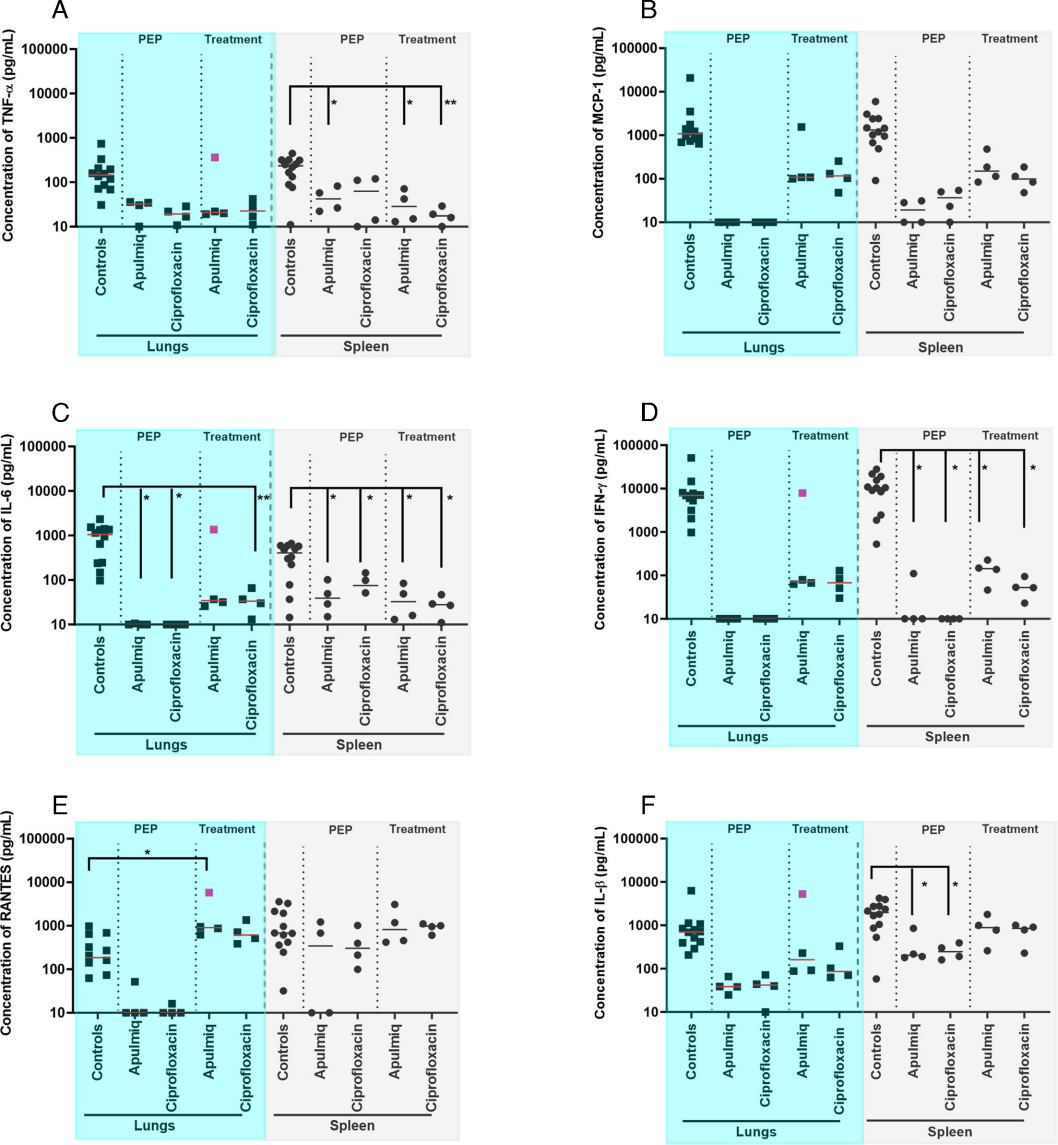
The level of selected cytokines in the lungs and spleens of animals that survived following challenge with *F. tularensis* by the inhalational route and administration of antibiotics (Apulmiq or ciprofloxacin) either as PEP (24 h post-challenge) or treatment (onset of fever). (**A**) TNF-a, (**B**) MCP-1, (**C**) IL-6, (**D**) IFN-g, (**E**) RANTES, (**F**) IL-1b. Significant difference from control animals (that received no antibiotic) was determined using a two-way ANOVA with Tukey’s multiple comparisons test, where * *P* = 0.05, ** *P* <0.01. The highlighted pink data point represents the animals that relapsed following treatment with Apulmiq. Control animals were euthanized when they reached the humane endpoint (between day 4 and 8 post-challenge), PEP and treatment animals were euthanized at the end of the study (day 21 and day 244, respectively).

The cytokine response was very similar in the lungs and spleens of these animals (Fig. 9). Animals that received oral ciprofloxacin or Apulmiq had lower levels of cytokines in these tissues than those that received no antibiotics, and those animals that were treated with antibiotics as PEP had lower cytokine levels than those that were treated on the onset of fever. The animal that received Apulmiq and had a secondary fever had comparable levels of TNF-α, IL-6, IFN-γ, RANTES, and IL-1β in the lungs to those animals that had received no antibiotics.

## DISCUSSION

The aim of this work was to determine whether delivery of ciprofloxacin by the inhalational route had any protective benefit over delivery by the oral route in the marmoset model of inhalational tularemia. All animals survived a lethal inhalational challenge of *F. tularensis* when ciprofloxacin was delivered by either route at 24 h post-challenge (PEP) or at the onset of fever (treatment). Although completely protected against infection, there was evidence of a potential resolving or recurring disease in most animals, and in one instance, an active, acute infection in the lungs. Minor interstitial pneumonia was observed in some of the animals that received Apulmiq or oral ciprofloxacin as PEP. The lesions were considerably less severe than the lesions observed in animals that did not receive antibiotics and could be suggestive of tissue healing. More evidence suggesting a potential resolution of infection was observed in the lungs of animals that received either Apulmiq or oral ciprofloxacin as treatment. Chronic lesions were observed with varying degrees of immune cell infiltration in at least one animal that received Apulmiq and one that received oral ciprofloxacin. Additionally, the cytokine response in the blood and lungs of animals that received either Apulmiq or oral ciprofloxacin as treatment was slightly raised, although it was approximately 10-fold lower than the levels observed in animals that did not receive antibiotics. Fluoroquinolones are known to reduce inflammation and potentiate the activity of the anti-inflammatory cytokine, IL-2 (28, 29). However, it is unlikely that this was a contributing factor to the lower cytokine response in treated animals, as one animal that received Apulmiq as treatment had higher levels of TNF-α, IL-6, IFN-γ, RANTES, and IL-1β in the blood and lungs. Encapsulated ciprofloxacin has, however, been shown to increase the activation of alveolar macrophages, which may increase bacterial clearance (26, 30). The animals with the raised cytokine response also had active lesions in the lungs, with bacteria detected by IHC and from plate counts. This was the only animal where there was any observable indication of disease, as they developed a secondary fever, despite showing no overt clinical signs.

The subsequent trajectory of the disease in the marmoset treated with the inhaled liposomal ciprofloxacin Apulmiq is somewhat uncertain based on the data collected, although it is likely that the disease was resolving in most animals except the animal that had a secondary fever, where the outcome is less predictable. In this study, marmosets were observed for 2 weeks following the cessation of a 7 day antibiotic regimen and were therefore euthanized on day 21 to day 24 post-challenge. In other studies, when cynomolgus macaques were challenged with inhalational *F. tularensis* and treated with antibiotic, animals were euthanized at 38 or 43 days post-challenge (8, 10). In those studies, fewer, although some, indicators of resolving disease were observed. Bacteria were recovered from the mediastinal lymph nodes of 3 out of 16 cynomolgus macaques treated for 21 days with TP-271 by the intravenous route (10). One animal had resolving, mild lesions in the lungs 43 days post-challenge following 10 days’ administration of gepotidacin by the intravenous route (8). A longer observation period in the current marmoset study would have helped to clarify whether these findings were analogous to those in the cynomolgus macaque study.

It is also worth noting that the 7 days administration of oral ciprofloxacin in the marmoset study was less than the 10 to 21 days recommended to treat human cases of tularemia (1) and was used to discriminate between ciprofloxacin administered by different delivery routes. Despite this, all animals treated with ciprofloxacin by either route survived. In humans, oral or intravenous ciprofloxacin has been used to successfully treat clinical cases, with some reports of good protection (31). However, relapse has been reported, particularly when treatment has been delayed (4, 5, 32). Ciprofloxacin may even be a better treatment for pneumonic disease, but even with treatment, the case fatality rate in humans is ~2%–3% for type A strains (4). Therefore, the fact that the inhalational dose of Apulmiq is 1.6% of the typical dose of oral ciprofloxacin administered indicates the benefit of delivering the antibiotic to the target site of infection. This is important for antibiotic stewardship; however, clinically, when treating infection caused by a highly virulent pathogen, this is less of a priority. Alternative fluoroquinolones, such as finafloxacin, levofloxacin, moxifloxacin, and gatifloxacin, have been shown to be effective in animal models (11, 33–35), although, with the exception of levofloxacin, they have not been used clinically in humans.

The cynomolgus macaque has been recommended as a suitable model of inhalational tularemia for use in studies to satisfy the FDA Animal Rule (36). Despite being a small, New World (platyrrhine) monkey, marmosets are a useful alternative to Old World (catarrhine) monkeys for studying tularemia (9, 37, 38). Marmosets that did not receive any antibiotic (control animals) exhibited features of tularemia previously reported in this model (9, 37). The pathological features observed, such as organ necrosis, thrombosis, and lung edema have also been reported in human cases of *F. tularensis* infection (39–41). The marmoset model is also highly comparable to the cynomolgus macaque. The mean time to fever in the marmoset is 64.3 h, and in the cynomolgus macaque, it was 57.7 ± 8.6 h, although the onset of clinical signs differed. Clinical signs in the cynomolgus macaque occurred concurrently with fever, while in the marmoset, they typically occur 24 h later. An increase in the levels of liver and kidney markers (AST, ALT, and BUN) is observed in both species, and the histopathological presentations are similar. The mean time to death for cynomolgus macaques is between 6 and 14 days, compared to between 4.9 and 7.7 days for the marmosets in this study, with a reproducible disease time course observed at ~300 to 3,000 CFU for cynomolgus macaques and 100 to 1,000 CFU for marmosets. Based on the primary endpoint (survival) and a secondary endpoint (fever), established for the cynomolgus macaques (36), ciprofloxacin administered by the oral or inhalational route in the marmosets was efficacious. The secondary, confirmatory endpoints recommended in the cynomolgus macaque are to ensure that there is appropriate disease progression. This was determined to be the onset of fever within 81 h and the presence of bacteremia (although this may not always be present). However, the additional immunological and IHC analyses performed in this marmoset study indicate the importance of considering other parameters, especially when comparing antibiotics.

The cynomolgus macaque was used to assess the efficacy of ciprofloxacin administered intravenously twice daily for 14 days against pneumonic tularemia. Treatment was initiated at 24 or 48 h after the onset of fever (13). Similar to the protection observed in the marmoset model, 100% survival was observed with intravenous administration of ciprofloxacin. In contrast, complete bacterial clearance was observed in all cynomolgus macaques by plate culture, although some animals remained PCR positive for *F. tularensis* in the blood up to day 29 post-challenge. This indicates that both the route of antibiotic delivery and the duration of treatment are important considerations when assessing efficacy.

In conclusion, this work demonstrates that inhalational ciprofloxacin (Apulmiq) offers protection against inhalational tularemia, although oral ciprofloxacin was more effective in this study. In addition, the common marmoset is a useful alternative nonhuman primate model to assess antimicrobials for this disease.

## MATERIALS AND METHODS

### Animals

Healthy, sexually mature common marmosets (*C. jacchus*) were obtained from the Dstl Porton Down breeding colony and housed in female and vasectomized male pairs. Animals were aged between 1.4 and 5 years old and weighed between 353 and 498 g at the start of the study, with no statistically significant difference in the ages or weights of the animals in the two studies. Two cohorts of animals were used: for the PEP study, a cohort of 16 animals was randomly allocated, in pairs, to four groups (no treatment, empty liposomes, Apulmiq, and oral ciprofloxacin). For the treatment study, a cohort of 12 animals was randomly allocated, in pairs, to three groups (Apulmiq, oral ciprofloxacin, and placebo) (see below for details). Blinding was undertaken as far as practical in the studies. However, staff involved in the delivery of the antibiotics (oral and inhalational) may also have been involved in determining the humane endpoint. To reduce individual bias, all decisions to euthanize animals were made in conjunction with a second person. All data analysis (bacteriology, immunology, and histology) was initially performed blinded, with the group allocations revealed for the final comparative analysis.

Diet and a variety of food were provided daily, and irradiated water was provided *ad libitum*. Animal rooms were illuminated with fluorescent lights and maintained on either a 12 h light/dark cycle (non-containment) or 14/10 h light/dark cycle (containment). Temperature was maintained between 23 and 28°C and a relative humidity between 40 and 70%. Environmental enrichment included deep litter foraging, various-sized Tupperware boxes, and textured perches. Prior to use in the efficacy studies, all animals were surgically implanted intraperitoneally, under general anesthesia (ketamine/medetomidine/isoflurane), with a Remo 201 device (EMMS, Bordon, Hampshire, UK) to record core body temperature (Tc) remotely. Prophylactic pain relief consisting of 0.2 mg/kg of meloxicam and 0.005 mg/kg of buprenorphine was administered. Data were analyzed using the eDacq software to provide real-time and recordable Tc (EMMS, Bordon, Hampshire, UK). At least 7 days prior to the challenge, blood was collected from all animals to assess the baseline levels of immunological markers. Animals were anesthetized with 5 mg of ketamine hydrochloride, and up to 2.5 mL of blood was collected from the femoral vein. Following the challenge, animals were observed at least three times a day, at 8-hourly intervals for 21 days. Clinical signs were recorded at least twice a day and included changes to posture, mobility, respiratory rate, fur condition, and behavior. Animals were weighed daily. Animals that succumbed to disease and those that survived until the end of the study were humanely euthanized, and blood and tissues (liver, spleen, kidney, lungs) were collected for further analysis (see below).

### Bacterial strain and culture

*F. tularensis* subsp. tularensis strain Schu S4 was streaked onto blood cysteine glucose agar (BCGA) with supplements and incubated for 24 h at 37°C. A loopful of bacteria taken from the plate was suspended in phosphate-buffered saline (PBS), adjusted to an OD_600_ of 0.1 (approximately 5 × 10^8^ CFU/mL), and used to inoculate 50 or 100 mL modified cysteine partial hydrolysate (MCPH) broth with 4% glucose to a final concentration of approximately 5 × 10^6^ CFU/L. Broths were incubated with shaking at 37°C for 48 h. The OD_600_ of the culture was adjusted to 0.1 and serially diluted to achieve the desired concentration for animal challenge. The CFU count used to infect the animals was determined by enumeration on BCGA plates. Blood was serially diluted in PBS and plated out onto BCGA plates. A section of animal tissue was weighed and homogenized in 5 mL of PBS using a cell strainer and plunger. The homogenate was serially diluted in PBS and plated out onto BCGA plates. Plates were incubated at 37°C for at least 48 h. Processing occurred within 2 h of tissue removal.

### MIC assays

MICs were conducted on individual bacterial colonies isolated from the lungs of one of the surviving marmosets using the Etest method in ambient air. Bacteria were resuspended into MCPH broth, which was adjusted to an OD_600nm_ of 0.1 (equivalent to ~1 × 10^8^ CFU/mL). A swab was streaked onto a BCGA plate, and the Etest was applied. The plates were incubated at 37°C, and the MIC was read following 48 h of incubation.

### Aerosol challenge

Aerosols of *F. tularensis* were generated using a three-jet Collison nebulizer and conditioned using an AeroMP (Aerosol Management Platform) aerosol system (Biaera Technologies L.L.C.). Marmosets were exposed to the aerosols for 10 min via a head-only exposure chamber, with sampling of the aerosol performed using an all-glass impinger (AGI-30; Ace Glass, NJ) containing PBS. An enumeration of *F. tularensis* in the aerosol was performed, and the total accumulated tidal volume for each animal during the challenge was determined by whole-body real-time plethysmography with a Fleisch pneumotachograph (EMMS, UK). The target challenge dose was 200 CFU, with the median lethal dose of *F. tularensis* in marmosets being less than 10 CFU (37), and the variability observed was due to the individual breathing rates of animals.

### Antibiotics

#### Preparation

Apulmiq was prepared by mixing equal volumes of Lipoquin (50 mg/mL) and free ciprofloxacin solution (20 mg/mL), both supplied by Aradigm Corporation (USA), to give a final concentration of 35 mg/mL. Empty liposomes were also provided by Aradigm Corporation and were used as supplied. For the orally administered ciprofloxacin, film-coated ciprofloxacin tablets (500 mg; Bayer UK) were dissolved in 25 mL of sterile-filtered water to give a final concentration of 20 mg/mL. The concentration of ciprofloxacin was adjusted in banana-flavored Nesquik powder reconstituted in distilled water, to deliver a final concentration of 50 mg/kg per animal. All antibiotics were prepared each day prior to dosing.

#### Antibiotic dosing regimen

Apulmiq was delivered by the inhalational route, as described below, to give a lung dose of 0.8 mg/kg once a day for 7 days (26), and ciprofloxacin (50 mg/kg) was administered twice daily for 7 days. This was based on pharmacokinetic modeling using a MIC of *F. tularensis* of 0.03 µg/mL and data previously generated in the marmoset (42). The antibiotic was administered at 24 h post-challenge (PEP) or at the onset of fever (treatment). Empty liposomes were delivered by the inhalational route once a day for 7 days, and placebo (banana-flavored Nesquik powder reconstituted in distilled water) was administered via the oral route twice a day for 7 days.

#### Inhalational delivery of antibiotics

Prior to dosing, animals were anesthetized using a reversal cocktail of fentanyl (0.01 mg/kg), medetomidine (0.06 mg/kg), and midazolam (0.5 mg/kg) delivered intramuscularly (26). The Apulmiq aerosol was generated using a Pari eFlow nebulizer, and marmosets were exposed to this using a bespoke marmoset inhalational therapy system via the attached head-only exposure chamber to deliver a lung dose of 0.8 mg/kg (26).

### Clinical chemistry

Blood was collected from animals pre-challenge and post-mortem into lithium heparin blood tubes. Plasma from heparinized blood was analyzed using a “dry-slide” technology biochemistry analyzer (Catalyst Dx, IDEXX).

### Histopathological analysis

Tissues were fixed in 10% neutral buffered formalin and processed for paraffin wax embedding using standard techniques. Thin sections (4 µm) were cut and stained with hematoxylin and eosin for histopathological analysis or Gram Twort to allow for visualization of bacteria. The tissues were examined by light microscopy and evaluated subjectively. The lesion profiles were evaluated and scored from 0 to 4 as follows: 0 = within normal limits, 1 = minimal changes, 2 = mild, 3 = moderate, and 4 = severe.

### Immunohistochemistry

Sections of tissue were stained for immunohistochemical detection of *F. tularensis* LPS antigen (FB11), macrophages and neutrophils (MAC387), T cells (CD3), or B lymphocytes (CD79a) (Table 3). Sections used for immunolabeling were dewaxed, dehydrated, and any endogenous peroxidase activity was quenched in 3% hydrogen peroxide in methanol for 15 min to eliminate activity prior to antigen retrieval. The samples were treated with trypsin/alpha-chymotrypsin (0.5% trypsin and 0.5% alpha-chymotrypsin; Sigma-Aldrich, Gillingham, Dorset, UK) at 37°C for 10 min. Sections were then microwaved in citric acid buffer (2.1 g citric acid; Fisher Scientific, Loughborough, Leicestershire, UK, in 1,000 mL distilled water), pH 6.0, for 18 min, 90% effect (780 W) or in Dako high pH 9.0 buffer (Dako UK Ltd.) for 10 min. The sections were then assembled into Sequenza coverplates (Shandon Scientific, Runcorn, UK) and rinsed with Tris-buffered saline (TBS), pH 7.6, 0.005 M (Sigma-Aldrich, USA). Primary antibody cross-reactivity with tissue constituents was prevented by using a 1.5% goat serum block in TBS for 20 min, followed by incubation with the primary antibody diluted in TBS for 1 h at room temperature. The sections were washed in TBS and incubated for 30 min with Dako REAL EnVision polymer (Dako UK Ltd.). The immunohistochemical signal was visualized using 3,3´-diaminobenzidine (Sigma-Aldrich), and sections were counterstained in Mayer’s hematoxylin (Surgipath, UK), dehydrated in absolute alcohol, cleared in xylene, and mounted using dibutyl phthalate xylene and glass coverslips. Positive and negative controls were used, and technique controls also included serial sections incubated with a protein of the same isotype immunoglobulin for each primary antibody, as well as omission of the primary antibody.

**TABLE 3:** Summary of primary antibodies and antigen retrieval methods used for immunohistochemistry

Antibody	Specificity	Source	Antigen retrieval method	Dilution
Mouse vs *F. tularensis* LPS, clone FB11	*F. tularensis* LPS	Invitrogen	Microwave, pH 9.0	1/2,000 and 1/4,000
Mouse vs human macrophages, clone MAC387	Monocytes, macrophages, and neutrophils	AbD Serotec	Trypsin-chymotrypsin	1/400
Rabbit vs human CD3	T lymphocytes	Dako	Trypsin-chymotrypsin	1/500
Mouse vs human CD79a	B lymphocytes	Dako	Microwave pH 6.0	1/100

### Cytokine analysis

Following the downstream processing of tissues harvested from marmosets, the plasma and supernatants collected from homogenized lung and spleen tissue were stored at −80°C. The levels of IL-6, IL-1β, MCP-1 (CCL2), and RANTES (CCL5) were determined using antibodies from the BD Cytometric Bead Array (CBA, BD Biosciences) human Flex set. The concentration of IFN-γ and TNF-α was determined using custom-made kits manufactured by BBI Detection Ltd (Salisbury, UK) using marmoset-specific antibodies produced by Mabtech (Stockholm, Sweden) and U-Cytech Biosciences (Utrecht, The Netherlands). Samples were analyzed on a BD FACSCanto II (BD Biosciences, Wokingham, UK), and the quantities of bound cytokines were determined using the BD FACSDiva software (BD Biosciences, Wokingham, UK).

### Antibody

The levels of anti-*Francisella* antibodies (IgM and IgG) in plasma were determined pre-challenge and at the end of the study by ELISA. Maxisorp ELISA plates (Thermo Scientific) were coated with 100 µL/well of 1 × 10^7^ /mL of heat-killed *F. tularensis* strain Schu S4 in carbonate coating buffer (Sigma, UK) and allowed to adhere overnight at 4°C. The plates were washed three times with PBS + 0.5% Tween, and blocked with 200 µL of block (2% skimmed milk powder in PBS + 0.5% Tween), for 2 h at room temperature. Plates were washed three times with PBS + 0.5% Tween, and 100 µL of plasma was added. This was performed in duplicate, at a starting dilution of 1:20, diluted fivefold across the plate, and incubated at room temperature for 2 h. Plates were washed three times with PBS + 0.5% Tween, and either 100 µL of anti-human IgG or IgM (labeled with horseradish peroxidase) (Sigma, UK) was added at a dilution of 1:1,000 and incubated at room temperature for 1 h. Plates were washed three times with PBS + 0.5% Tween, and a color change was observed via a plate reader with 3,3´5,5´-tetramethylbenzidine and stop solution (Sigma, UK).

### Statistics

Comparative analysis of age, body weight, bacteriology, cytokine analysis, and liver enzyme data was performed using ANOVA or Kruskal-Wallis analysis, with some data transformed by log_10_ to ensure a normal distribution as appropriate.
